# Supplementary material for: Omega-3 fatty acid synergy with glucocorticoid in mouse lupus macrophage model: targeting pathogenic pathways to reduce steroid dependence
Source: Front Immunol. 2025 Oct 15;16:1646133. doi: 10.3389/fimmu.2025.1646133 (PMC12568668; doi:10.3389/fimmu.2025.1646133)
Supplement: Supplementary file 1 [file DataSheet1.docx]

Supplementary Material

Omega-3 Fatty Acid Synergy with Glucocorticoid in Mouse Lupus Macrophage Model: Targeting Pathogenic Pathways to Reduce Steroid Dependence

Lauren K. Heine^1,2†^, Rance Nault^1,2†^, Jalen Jackson^3^, Ashley N. Anderson^3^, Jack R. Harkema^1,2,4^, Andrew J. Olive^3^, James J. Pestka^2,3,5*^, Olivia F. McDonald^1,2,3*^

^1^Department of Pharmacology and Toxicology, Michigan State University, East Lansing, MI, United States

^2^Institute for Integrative Toxicology, Michigan State University, East Lansing, MI, United States

^3^Department of Microbiology, Genetics, and Immunology, Michigan State University, East Lansing, MI, United States

^4^Department of Pathobiology and Diagnostic Investigation, Michigan State University, East Lansing, MI, United States

^5^Department of Food Science and Human Nutrition, Michigan State University, East Lansing, MI, United States

***Correspondence:**
Dr. Olivia McDonald
favoroli@msu.edu

Dr. James Pestka
pestka@msu.edu

^†^These authors contributed equally to this work and shared the first authorship
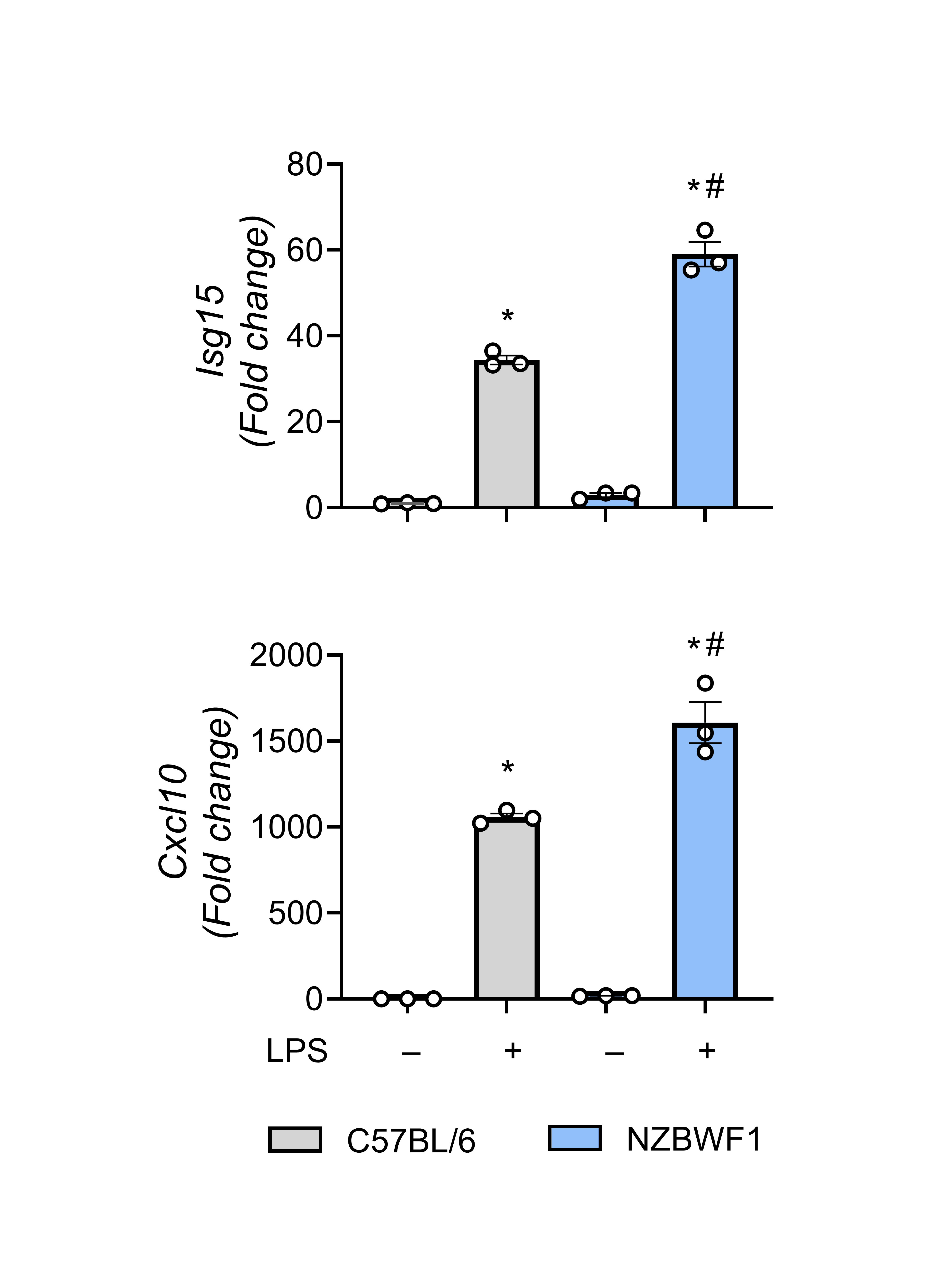


**Supplemental Figure 1.** **LPS-induced type I IFN gene responses are significantly elevated in SLE-prone NZBWF1 FLAMs compared to non-SLE C57BL/6 FLAMs**. qRT-PCR was performed on FLAMs stimulated with LPS (20 ng/mL) for 4 hr. Fold change is shown as LPS treatment relative to VEH ± SEM. n=3 biological replicates. p<0.05; *Significant compared to VEH; #Significant compared to C57BL/6 FLAM response.

**
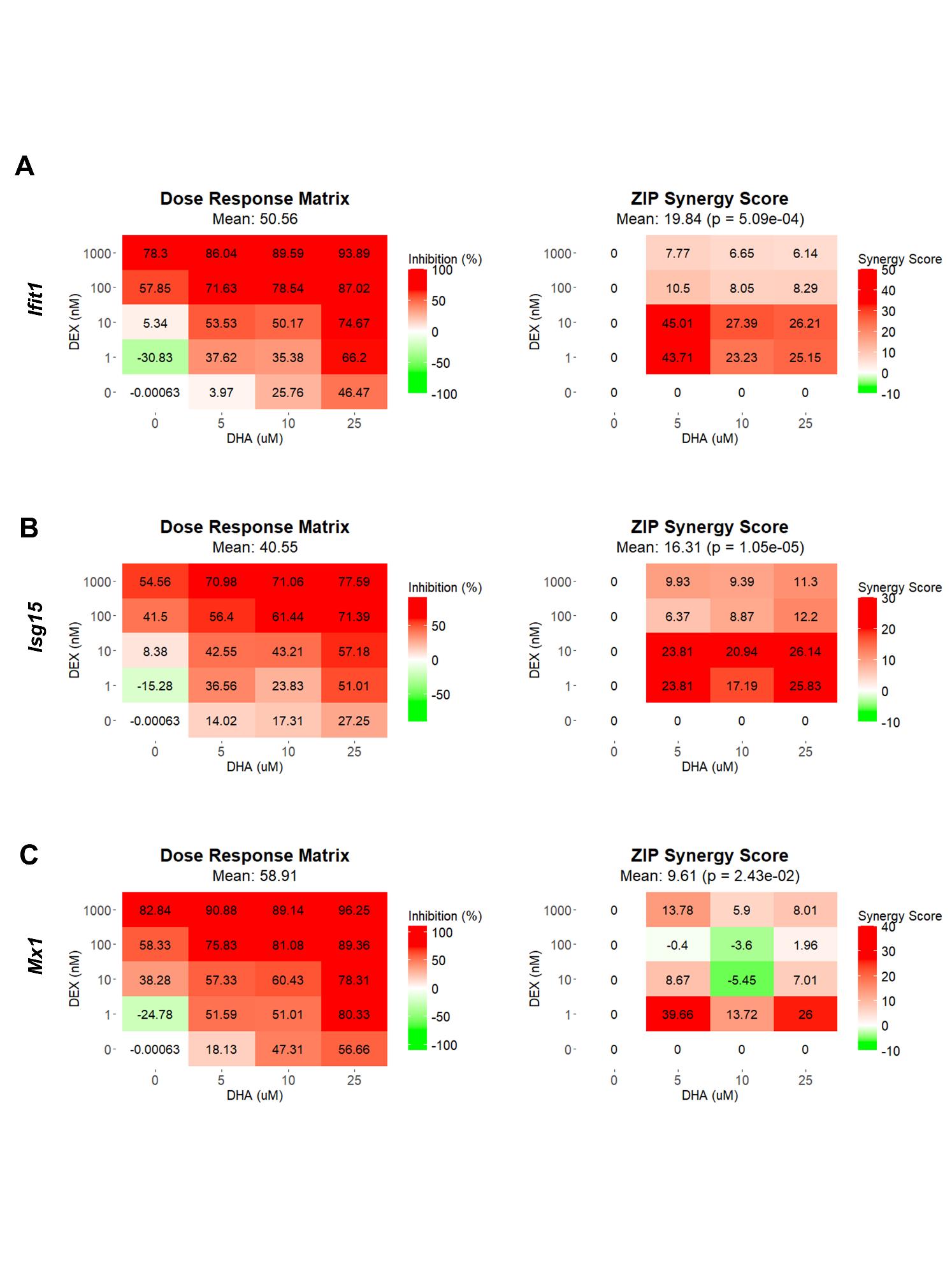
**

**Supplemental Figure 2. DHA and DEX synergistically inhibit the expression of IFN-stimulated genes.** *Ifit1* **(A)**, *Isg15* **(B)**, and *Mx1* **(C)** were measured by qRT-PCR in FLAMs stimulated with LPS (20 ng/mL) for 4 hr. Cells were pretreated with either VEH containing no DHA or RPMI media containing 25 µM, 10 µM, or 5 µM DHA at -24 hr. Cells were then treated with VEH containing no DEX or varying concentrations of DEX (1 nM-1 µM) -1 hr prior to LPS treatment. SynergyFinder version 3.14.0 was used to generate inhibition matrices and ZIP synergy matrices for each gene. Inhibition matrices show the average of 3 experimental replicates. Individual and mean ZIP synergy scores were calculated using an average of 3 experimental replicates. Synergy score > 0, synergistic interaction; synergy score = 0, additive effect; synergy score < 0, antagonistic interaction.

**
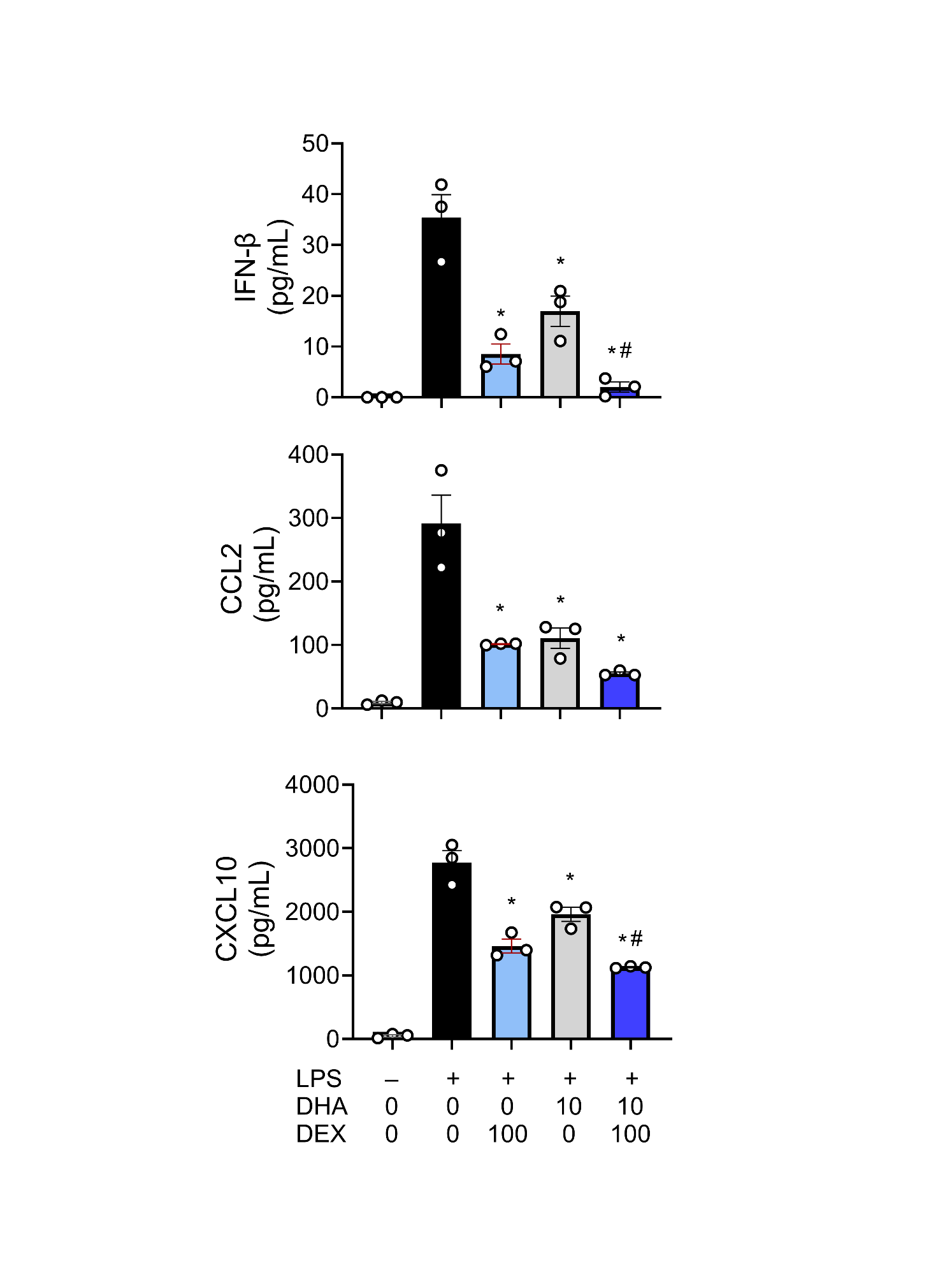
**

**Supplemental Figure 3. DHA+DEX combination treatment suppresses type I IFN-related protein secretion.** Cells were pretreated with either VEH containing no DHA or RPMI media containing 10 µM or 25 µM DHA at -24 hr. Cells were then treated with VEH containing no DEX or RPMI media containing 100 nM or 1000 nM DEX -1 hr prior to LPS treatment. Type I IFN-related proteins (i.e., IFN-β, CCL2, CXCL10) were measured by ELISA in supernatants from FLAMs stimulated with LPS (20 ng/mL) for 24 hr. Data are shown as mean ± SEM. n=3 biological replicates. p<0.05; *Significant compared to LPS/VEH; #Significant compared to DHA alone; †Significant compared to DEX alone.


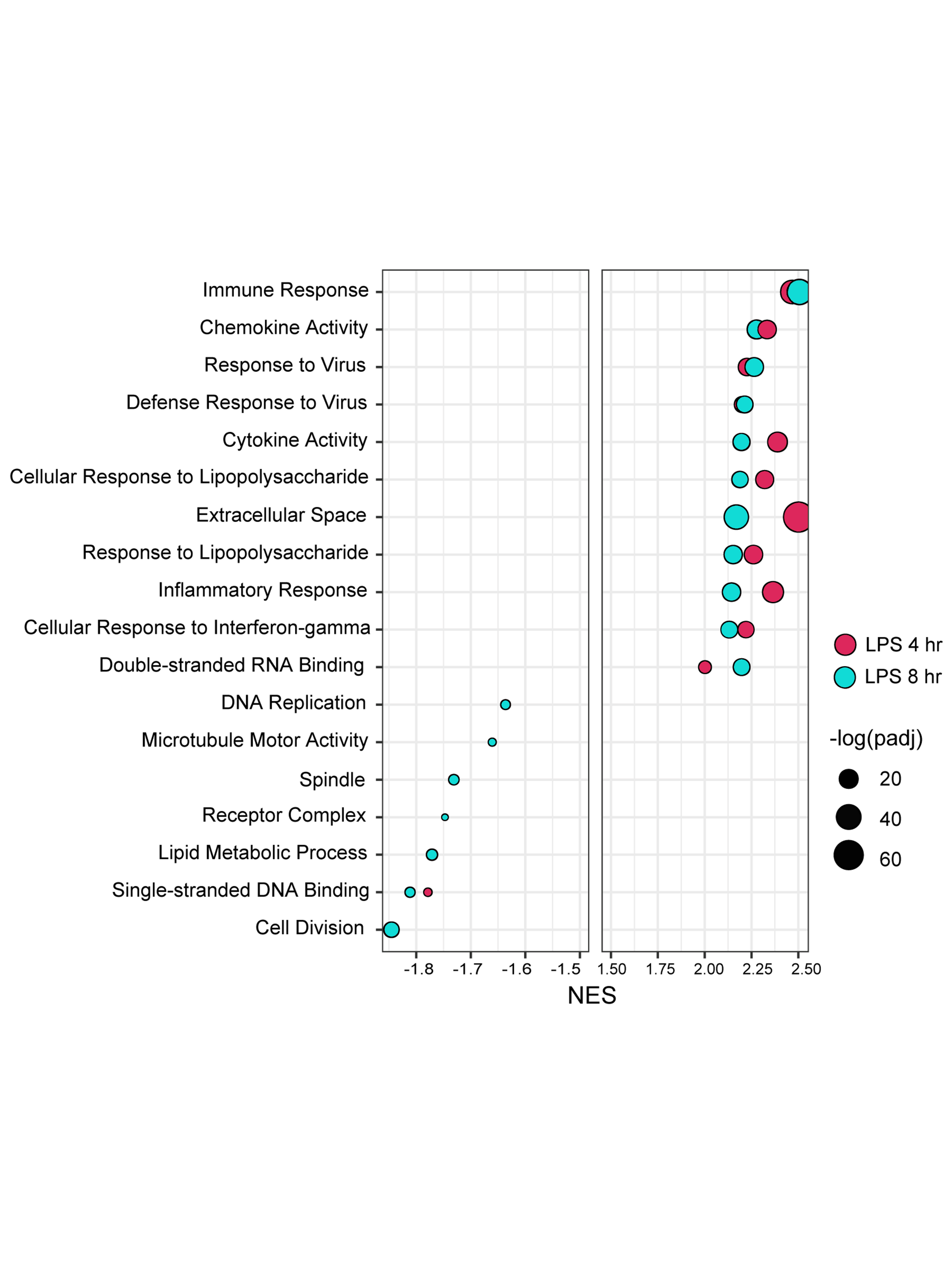


**Supplemental Figure 4. LPS induces inflammation and innate immunity and suppresses proliferative and metabolic biological processes.** Gene set enrichment analysis was performed using the fgsea package in R on gene expression datasets ranked by fold-change and gene sets from the Gene Set Knowledgebase (GSKB) (40) filtered only to include Gene Ontology (GO) and KEGG gene sets (41). At both 4-hr and 8-hr time points, LPS induced immune and inflammatory pathways, including cytokine/chemokine signaling and responses to viruses, LPS, IFN, and dsDNA. These immune-related processes showed equal or greater activation at 4 hr compared to 8 hr, highlighting the rapid and dynamic nature of the inflammatory response. Simultaneously, pathways related to cell proliferation and metabolism (blue bars) exhibited significant negative enrichment, including cell division, DNA replication, lipid metabolism, and microtubule-associated processes, indicating coordinated suppression of growth and metabolic functions during LPS-induced inflammation.
